# Quality of life of men and women with gender identity disorder

**DOI:** 10.1186/s12955-018-0995-7

**Published:** 2018-08-20

**Authors:** Banafsheh Torkian Valashany, Mohsen Janghorbani

**Affiliations:** School of Public Health, Isfahan University of Medical Sciences, Isfahan, Iran

**Keywords:** Quality of life, Gender identity disorder, Gender dysphoria, Hormone therapy, Sex reassignment, Male to female transgender, Female to male transgender

## Abstract

**Background:**

The aim of this study was to evaluate the self-reported perceived quality of life (QoL) in female to male (FTM) and male to female (MTF) transgenders and compare it with a general population sample, and to find possible determinants that likely contribute to their QoL.

**Methods:**

Participants were 71 trandgenders participating in the communities of Isfahan and Fars provinces, Iran, including 30 MTF and 41 FTM, and 142 gender- and age-matched controls. Persian version of the Short Form 36-Item Questionnaire was used to evaluate self-reported QoL, which measures QoL across eight domains.

**Results:**

Compared to control group, the QoL of transgenders in the most dimensions of the SF-36 questionnaire was lower. MTF had a lower QoL than FTM for the subscale physical functioning (*p* = 0.044). There was a significant relationship between education and subscales of emotional well-being (*p* = 0.048) and social function (*p* = 0.008); economic status and physical function subscale (*p* = 0.003); employment status and physical function (*p* = 0.012) and social function subscales (p = 0.003). Compared to male controls, MTF transgenders had lower physical functioning (*P* < 0.001), role limitation due to physical health (*P* = 0.015), vitality (*P* = 0.023), social functioning (P < 0.001) and pain score (*P* = 0.044) and no significant differences between female controls and FTM transgenders were seen.

**Conclusion:**

Transgenders have lower physical and mental QoL, FTM transgender has better QoL than MTF transgender. Employment, education, province of residence and economic status as well as therapeutic intervention is associated with transgender’s QoL.

## Background

Transgender people have a gender identity or expression that differs from their sex assigned at birth. Most individual’s gender identity is made until the age of 2 to 3 and it is harmonized to their biological gender [1]. Compared to the general population, many transgender people experience a higher distress related to the discrepancy between their birth genders and felt a sense of being male, female, or otherwise gender nonconforming [2, 3]. Gender identity disorder is generally accompanied by dissatisfaction with physical appearance, and a negative body image has been shown to be more prevalent among transgender women than transgender men [4].

Although the prevalence of gender identity disorder appears to be increasing worldwide [5], transsexuality phenomenon is not a common phenomenon [6]. In general, the prevalence of gender identity disorder is different in countries and it may be more common from what is imagined. The prevalence of this disorder is about one to three in one hundred thousand persons and ratio of men to women is about 3-5 to 1 [7]. However, in Iran it is close to 1 to 1 [8]. The prevalence of gender identity disorder in the Netherlands based on population data was 0.6% in men and 0.2% in women showing that estimation of the prevalence of the disease by referring patients to receive medical care is underestimated [9]. It can be predicted that in Iran there are about four thousand people with gender identity disorder [10].

Transgenders encounter with discrimination at individual, organizational and systemic levels [11]. In fact, in some transgender adolescents mental health problems, suicide and social isolation are formed [12]. People are in the danger of several mental-social difficulties containing rejection from the family and peers, persecution and harassment, trauma, abuse, inappropriate housing, lack of financial protection, training and legal difficulties [5]. Acute damage and depression, relieves it and relief and so they recovery the quality of life (QoL) [12].

There is a lack of consensus in the QoL of transgenders. A few previous studies have indicated that transgenders also experience lower QoL than the general population [13–15]. Conversely, other studies have found no difference in QoL or psychological functioning between transgenders and the general population [16–19].

Therefore, the aims of this study were first to assess QoL of MTF and FTM transgenders and compare with general population sample; second, to examine the differences between transgender men and women, and third; how their level of QoL differs according to socio-demographic factors. This information would be useful for future comparison of secular trend and to provide data on the OoL of Iranian transgenders, where such studies have not been carried out.

## Methods

### Subjects

This is a case-control study. All of the individuals with gender identity disorder with definite diagnosis using DSM-V-TR diagnostic criteria and being member in societies of people with gender identity disorder in Isfahan and Fars provinces (71 members) formed the case group. The cases include 4 groups: treated with hormones (*n* = 16), no hormone treatment (*n* = 55), undergoing sex reassignment surgery (*n* = 9), and no gender reassignment surgery (*n* = 62). Individuals were excluded from the study if they had an inability to respond or unwillingness to participate in the study and having severe psychiatric disorders or any limiting chronic diseases. Face to face interviews were done by a trained person. The data were processed and analyzed by authorized personnel only, and was anonymized and de-identified prior to analysis. The group of transgenders was compared with a control group of 142 healthy adults. Healthy controls were volunteers referred to counseling centers for getting educational counseling for children, family, job, etc. For each person who has a gender identity disorder two age-matched controls were considered.

### Ethics statement

This study approved by the Isfahan University of Medical Sciences ethical committee, and an informed consent form was signed by each participant.

### Data collection

The following variables were acquired by an interview and a self-administered questionnaire: age, gender, educational level (not having graduated, high-school diploma, or high education and holding a university degree), employment status, self-estimation of the financial situation (good, average, bad), relationship status (single vs. in a relationship), life entourages (those who already live in the same place with an involved person), the current use of sex hormone treatment, and having undergone one or more transition-related surgery.

### Main outcome measures

To measure QoL of individuals with and without transgender identity disorder, we used the Persian validated version of the self-reported physical and mental health Short Form-36 (SF-36) Health Survy [20]. This questionnaire has 36 independent questions and cover eight emotional and physical domains: physical function (evaluates the presence and severity of limitations to physical activities), role limitations due to physical health problems (assess the limitations to work or other daily activities), pain (determine the impact pain on daily activities), general health perceptions (determine overall sense of well-being), vitality (evaluates the influence of health on energy level and fatigue), social functioning (measures the impact of health on engaging in social activities), role limitations due to emotional problems (assess the impact of emotional problems performing daily activities) and general mental health (evaluates the presence or severity of mental health indicators; like anxiety and depression). The scores of each domain vary from zero to 100 which higher scores indicating higher levels of functioning or well-being. A short 36–item questionnaire was assessed by case group that were appropriate diagnosis to this research data and control group. Finally, to better understand the QoL of people ever sub-scale at any desired levels favorable (67–100), relatively favorable (33-66) and unfavorable (0–32) were analyzed and classified. By using the socio-demographic questionnaire (age, gender, occupation, housing status, marital status, education level, income, life entourages (those who already live in the same place with an involved person)) the related information to demographic variables were collected. Medical information containing the sex reassignment surgery, types of surgery, hormone therapy, duration of hormone therapy, and time elapsed since the surgery was collected by interview.

### Statistical analysis

Continuous and categorical variables are expressed as means with standard deviation (SD) or 95% confidence intervals (CIs) and percentages, respectively, unless otherwise specified. Distribution of data was checked for normality using the Kolmogrov-Simironov test. Statistical methods used comprised the t-test and one-way analysis of variance (ANOVA) to study the difference in QoL between MTF and FTM transgenders and QoL scores for MTF and FTM with a sample of healthy population. Because we conducted multiple statistical tests, we used the Bonferroni correction for multiple comparisons and correct for an increased chance of type I error. Covariate analyses adjusting for age were performed to compare differences between men and women. All tests for statistical significance were two-tailed, and all were done assuming a type I error probability of < 0.05. The SPSS software version 20 for Windows were used for data analysis.

## Results

### Characteristics

Differences in the distribution of several socio-demographic characteristics among 31 MTF and 41 FTM transgenders are shown in Table 1. MTF transgenders had higher education and were less likely to be married than FTM transgenders. The mean (SD) age was 23.8 (5.6) years for MTF and 24.2 (6.3) years for FTM transgenders.

**Table 1:** Demographic and clinical comparison of male-to-female and female-to- male transgenders

Characteristics	MTF (30)	FTM (41)	Difference (95% CI)
Mean age (SD)	23.8 (5.6)	24.2 (6.3)	−0.4 (−3.3, 2.5)
Mean term hormone therapy (SD)	2.7 (9.6)	4.8 (13.0)	−2.1 (− 7.7, 3.5)
Mean long after surgery (SD)	2.2 (8.4)	4.8 (13.2)	− 2.6 (−8.1, 2.9)
Hormone therapy			
Yes (%)	6 (20)	10 (24.4)	−4.4 (−23.8, 15.0)
No (%)	24 (80)	31 (75.6)	–
Surgery			
Yes (%)	2 (6.7)	7 (17.1)	−10.4 (−25.0, 4.2)
No (%)	28 (93.4)	34 (82.9)	–
Surgery type			
Upper (%)	0	1 (2.4)	−2.4 (−7.2, 2.3)
Lower (%)	1 (3.3)	1 (2.4)	0.9 (−7.1, 8.9)
Both (%)	1 (3.3)	5 (12.2)	−8.9 (−20.8, 3.0)
None (%)	28 (93.4)	34 (82.9)	10.4 (−4.2, 25.0)
Marriage			
Married (%)	0	1 (2.4)	−2.4 (−7.2, 2.3)
Single (%)	29 (96.7)	34 (82.9)	13.7 (0.6, 26.9)*
Divorced (%)	1 (3.3)	6 (14.6)	−11.3 (−23.9, 1.3)
Education			
Under high-school diploma (%)	5 (16.7)	6 (14.6)	2.0 (−15.1, 19.2)
High-school diploma (%)	13 (43.3)	17 (41.5)	1.9 (−21.4, 25.1)
Collegiate (%)	18 (43.9)	5 (33.3)	47.8 (27.6, 68.0)**
Economic status			
Poor (%)	8 (26.7)	6 (14.6)	12.0 (−7.1, 31.2)
Fair (%)	14 (46.7)	21 (51.2)	−4.6 (−28.1, 19.0)
Good (%)	8 (26.7)	14 (34.1)	−7.5 (− 29.0, 14.0)
Occupation			
Housewives (%)	3 (10)	0	10.0 (−0.7, 20.7)
Unemployed (%)	8 (26.7)	9 (22)	4.7 (−15.6, 25.0)
Employee (%)	4 (13.3)	1 (2.4)	10.9 (−2.2, 23.9)
Self-employed (%)	5 (16.7)	17 (41.5)	−24.8 (− 44.9, −4.7)
Other (%)	10 (33.3)	14 (34.1)	0.8 (−23.1, 21.4)
Address			
Urban (%)	29 (96.7)	38 (92.7)	4.0 (−6.3, 14.2)
Rural (%)	1 (3.3)	3 (7.3)	–
Housing			
Owner-occupier (%)	1 (3.3)	2 (4.9)	−1.5 9 (−10.8, 7.7)
Leased (%)	1 (3.3)	3 (7.3)	−4.0 (−14.2, 6.3)
Parent home (%)	28 (93.4)	35 (85.4)	8.0 (−6.1, 22.0)
Other (%)	0	1 (2.4)	−2.4 (−7.2, 2.3)
Entourage			
Father (%)	2 (6.7)	2 (4.9)	1.8 (−9.3, 12.9)
Mother (%)	1 (3.3)	4 (9.8)	−6.4 (−17.5, 4.7)
Parents (%)	25 (83.3)	27 (65.9)	17.5 (−2.2, 37.2)
Partner (%)	0	1 (2.4)	−2.4 (−7.2, 2.3)
Partner and child (%)	0	2 (4.9)	−4.9 (−11.5, 1.7)
Lonely (%)	1 (3.3)	3 (7.3)	−4.0 (−14.2, 6.3)
Other (%)	1 (3.3)	2 (4.9)	−1.5 (− 10.8, 7.7)
Province			
Isfahan	16 (53.3)	26 (63.4)	−10.1 (−33.2, 13.1)
Fars	14 (46.7)	15 (36.6)	–

*p < 0.001, **p < 0.05

Characteristics of the 71 transgenders and 142 controls are shown in Table 2. Transgenders were poorer, and less likely to be married than controls.

**Table 2:** Comparison of the socio-demographic characteristics between case and control groups

socio-demographic characteristics	Case group (71)	Control group (142)	Difference (95% CI)
Mean age (SD)	24.0 (6.0)	25.2 (6.8)	− 1.2 (−3.1, 0.7)
Marriage			
Married (%)	1 (1.4)	87 (61.3)	− 59.9 (−68,3, −51.4)**
Single (%)	63 (88.7)	55 (38.7)	50.0 (39.1, 60.9)**
Divorced (%)	7 (9.9)	0 (0.0)	9.9 (2.9, 16.8)**
Education			
Under high-school diploma (%)	11 (15.4)	30 (21.1)	−5.6 (−16.4, 5.1)
Hi-school diploma (%)	30 (42.3)	45 (31.7)	10.6 (−3.2, 24.4)
Collegiate (%)	30 (42.3)	67 (47.2)	−4.9 (−19.1, 9.2)
Economic status			
Poor (%)	14 (19.7)	7 (4.9)	14.8 (4.9, 24.7)*
Fair (%)	35 (49.3)	94 (66.2)	−16.9(−30.9, −2.9)*
Good (%)	20 (28.2)	37 (26.1)	2.1 (−10.6, 14.8)
Excellent (%)	2 (2.8)	4 (2.8)	0.0 (− 4.7, 4.7)
Occupation			
Housewives (%)	3 (4.2)	49 (34.5)	−30.3 (−39.4, 21.2)**
Unemployed (%)	17 (23.9)	9 (6.3)	17.6 (6.9, 28.3)**
Employee (%)	5 (7.0)	22 (15.5)	8.5 (−16.9, 00.03)*
Self-employed (%)	22 (31.0)	21 (14.8)	16.2 (4.0, 28.4)**
Other (%)	24 (33.8)	41 (28.9)	4.9 (−8.4, 18.2)
Address			
Urban (%)	67 (94.4)	126 (88.7)	5.6 (−1.8, 13.1)
Rural (%)	4 (5.6)	16 (11.3)	–
Housing			
Owner-occupier (%)	3 (4.2)	40 (28.2)	−23.9 (−32.7, −15.2)**
Leased (%)	4 (5.6)	39 (27.4)	−21.8 (−30.9, −12.7)**
Parent home (%)	37 (52.1)	63 (44.4)	7.8 (−6.5, 22.0)
Other (%)	1 (1.4)	0 (0.0)	1.4 (−1.3, 4.2)
Entourage			
Father (%)	4 (5.6)	0 (0.0)	5.6 (0.3, 11.0)*
Mother (%)	5 (7)	2 (1.4)	5.6 (−0.6, 11.9)
Parents (%)	52 (73.2)	62 (43.7)	29.6 (16.4, 42.7)**
Partner (%)	1 (1.4)	23 (16.2)	−14.8 (−21.4, −8.1)*
Partner and child (%)	0 (0.0)	55 (38.7)	−38.7 (−46.7, − 30.7)**
Lonely (%)	4 (5.6)	0 (0.0)	5.6 (0.3, 11.0)*
Other (%)	3 (4.2)	0 (0.0)	4.2 (−0.5, 8.9)

*p < 0.001, **p < 0.05

### Perceived QoL

Compared to the control group, transgeders rated their QoL significantly lower in the dimensions, physical functioning (*p* < 0.001), social functioning (p < 0.001) and role limitations due to physical function (*p* = 0.035), and vitality (*p* = 0.017) (Table 3). Compared with male controls, MTF transgenders had lower physical functioning (*P* < 0.001), role limitation due to physical health (*P* = 0.015), vitality (*P* = 0.023), social functioning (P < 0.001) and pain score (*P* = 0.044) (Table 3). No significant differences between female controls and FTM transgenders were seen.

**Table 3:** Comparison of the following sub-scales of quality of life scores for male and female transgender with the control group

	Mean (SD)		
	MTF transgender	Male controls	Difference (95% CI)
Physical functioning	74.8 (16.1)	95.6 (16.8)	−20.8 (− 28.0, −13.6)*
Role physical	61.7 (27.6)	83.6 (25.5)	−21.9 (−33.2, −10.6)**
Role emotional	64.4 (34.9)	74.7 (30.1)	−10.3 (−23.9, 3.4)
Vitality	54.5 (19.9)	68.1 (16.6)	−13.6 (−21.2, −6.0)**
Emotional well-being	61.7 (18.1)	71.5 (16.7)	−9.8 (−17.2, −2.4)
Social functioning	56.3 (26.2)	80.4 (15.7)	−24.1 (−32.5, −15.7)**
Pain	74.3 (23.0)	88.2 (14.2)	−13.9 (−21.4, −6.5)*
General health	57.3 (22.7)	62.1 (15.6)	−4.8 (−12.6, 3.0)
	FTM transgender	Female control	
Physical functioning	85.5 (15.9)	84.6 (19.3)	0.9 (−6.2, 8.0)
Role physical	70.7 (27.9)	71.1 (33.3)	−0.4 (−12.6, 11.8)
Role emotional	62.2 (20.9)	70.6 (36.4)	−8.4 (−20.7, 3.9)
Vitality	59.0 (22.8)	62.4 (19.0)	−3.4 (−11.4, 4.6)
Emotional well-being	67.4 (18.5)	67.0 (18.4)	0.4 (−6.8, 7.6)
Social functioning	68.0 (20.9)	71.4 (21.7)	−3.4 (−11.7, 4.9)
Pain	78.1 (23.8)	74.2 (23.6)	3.9 (−5.3, 13.1)
General health	69.6 (20.4)	67.7 (19.7)	1.9 (− 5.9, 9.7)

*p < 0.001, **p < 0.05

MTF and FTM transgender in any of the subscales except physical functioning (*p* = 0.044), social functioning (*p* = 0.047) and general health (*p* = 0.041) subscales have no significant difference (Table 4).

**Table 4:** Comparison of quality of life for male-to female and female-to male transgender

	Mean (SD)		
	MTF Transgender	FTM transgender	Difference (95% CI)
Physical functioning	74.8 (16.1)	85.5 (15.9)	−10.7 (− 18.4, −3.0)*
Role physical	61.7 (27.6)	70.7 (27.9)	−9.0 (− 22.3, 4.3)
Role emotional	64.4 (34.9)	62.2 (20.9)	2.2 (−11.1, 15.5)
Vitality	54.5 (19.9)	59.0 (22.8)	4.5 (−14.9, 5.9)
Emotional well-being	61.7 (18.1)	67.4 (18.5)	−5.7 (−14.5, 3.1)
Social functioning	56.3 (26.2)	68.0 (20.9)	−11.7 (−22.9, −0.5)*
Pain	74.3 (23.0)	78.1 (23.8)	−3.8 (− 15., 7.5)
General health	57.3 (22.7)	69.6 (20.4)	−12.3 (− 22.6, − 2.0)*

*p < 0.05

### Medical characteristics

In MTF transgenders there was a significant association between hormone therapy and sub-scales of the physical role limitations (*p* = 0.008), emotional health (*p* = 0.048) and social functioning (p = 0.048) and in FTM transgender with subscales of vitality (*p* = 0.002), emotional health (*p* < 0.001), social functioning (p = 0.002), and pain (*p* = 0.033). After controlling the duration of hormone therapy, hormone therapy in MTF has only been related to the subscale of physical role limitations (*p* = 0.021) and in the FTM merely with emotional health subscale (*p* = 0.039).

There was a significant positive relationship between surgical intervention in MTF in subscales of physical functioning (*p* = 0.037), vitality (*p* = 0.012), emotional health (*p* = 0.002), social functioning (p = 0.012) and in FTM in the subscales of the physical role limitations (*p* = 0.019), vitality (*p* = 0.003), emotional health (p = 0.003), social functioning (*p* < 0.001), and pain (*p* = 0.005). After controlling the duration of surgery, surgical intervention was not significantly associated with any subscales of QoL in both MTF and FTM transgenders.

There was a significant relationship between the type of surgery in MTF with sub-scales of social functioning (*p* = 0.045) and in FTM with subscales of vitality (*p* = 0.016), emotional health (*p* = 0.025), social functioning (*p* = 0.008) and general health (*p* = 0.028).

### Sociodemographic indicators and QoL in transgender group

There is a positive correlation between age (*p* = 0.009), healthy emotional (*p* = 0.003) and social functioning (*p* = 0.011) and a significant difference between education and well-being (*p* = 0.048) and social functioning subscales (*p* = 0.008), also a significant difference between economic status and physical function subscale (*p* = 0.003) and between employment status and physical functioning (*p* = 0.012) and social functioning subscales (p = 0.003) is seen and students have lowest QoL. Transgenders living in Fars province in the domains of well-being (*p* = 0.042), social functioning (*p* = 0.023) have better QoL than transgenders living in Isfahan province.

## Discussion

Transgenders reported a lower QoL in the most dimensions of the SF-36 questionnaire, than the control group. MTF transgenders had a lower QoL than FTM transgenders for the subscale physical functioning. Hormone use was positively associated with subscales role limitations due to physical function in MTF and with subscale emotional health in FTM transgenders. As expected, education was positively associated with QoL of emotional well-being and social function and economic status. There are a positive association between employment and physical and social function subscales.

This study did not demonstrate any influence of surgical intervention on QoL. A small number of transgenders has undergone surgery or hormonal intervention. These people usually after receiving therapeutic interventions, especially surgical intervention, left their homes and moved to a new location and a new gender of them is recognized, thus rarely and only shortly after intervention get to participate in meetings and prefer to leave the community and to forget the past and resolved in the course of normal life, therefore, these interventions was not considered for comparison.

Compared with the control group, QoL of transgenders was lower in physical function subscale. These results are similar to the results of the study of Gurion- Lazard et al. [21] but in contrast to the results of the study of Newfield et al. [15] that indicated physical function subscale has better scores than the control group.

In this study, the FTM transgenders had better QoL than MTF transgenders only in the physical functioning subscale. But, in overall they had higher QoL scores. Reisner et al. reported that there is a significant difference between transgender males and females in mental health outcomes [22]. Although the more MTF transgenders in this study are similar to men in terms of physical condition and still do not receive hormone or surgical intervention, but they have similar QoL with women in all subscales of QoL, except role limitations due to emotional problems, general health, and emotional well-being. Motman et al. [23] and De Verise et al. [24] showed that there was no significant difference between transgender groups in none of the QoL subscales and women in the society. The study findings of Newfield et al. also indicated that FTM transgenders had the QoL similar to women [15]. Motman et al. have shown that MTF transgenders compared with the sample of Dutch society, men received lower scores on the subscales vitality and mental health and in line with the results of this study [23]. According to the country’s cultural status and desire to have male children in most parts of the country, women who seek to change gender gain higher culture points and the more family and community acceptance. Our findings on lower QoL in MTF compared to FTM is in line with the results of many studies carried out in other parts of the world [23, 25, 26] and in contrast to a few others [27].

By controlling the duration of hormone therapy, hormone therapy in MTF transgenders was associated only with the subscales of physical role limitations and emotional well-being in FTM. This study shows that long-term hormone therapy had strongly linked with subscales of QoL. Newfield et al. indicated that after 15 years of hormone therapy, hormone therapy associated only with physical role limitations and physical function subscales [15]. However, Gomez Gil et al. [28] and De Vries et al. [24] showed that hormone therapy is strongly associated with QoL. Fuss et al. [29] also reported that after 12 months of hormone therapy in the MTF, there was no change in levels of physical activity, but there was sense of general health and create meaningful change.

By controlling the duration of surgery, surgical intervention in transgenders had no significant relationship with any of the subscales of QoL that is in line with several studies [24, 28, 30, 31] and antithetical to the results of other studies [23, 25, 27, 32]. Transgender people who had both upper and lower sex reassignment surgery had a better QoL than the control group. The results were similar to the other studies [25, 33, 34].

Unlike some studies [15, 28], in the transgender group the age has a positive correlation with the scale of vitality, emotional health and social functioning and overall scale of the physical and mental health. The case group was young and a significant number of them were under 20 years. These people were at puberty stage and problems associated with this stage of life along with gender identity disorder and disgust with current physical condition, is justified positive correlation between age and the subscales. These results are similar to results obtained from the study of Barila et al. [35].

Transgenders with higher education and better socioeconomic status experienced a better QoL. Those with lower education and socioeconomic status encountered more problems, including obtaining jobs or follow-up treatment due to its high cost, and the combination of these factors may severely affect QoL especially on the mental health aspect. Lack of family and community support will complicate the situation. Gomez Gil et al. [28] showed that education is not associated with any of the QoL subscales and inconsistent with the results of this study, but larger studies have confirmed the relationship between education and QoL subscales [21, 23, 24, 27]. Transgender students have lower QoL scores than those with self-employed. Fear and worry about not having a job, rejection by friends, peers and family and uncertain future probability are factors that have an effect on QoL in physical and social functioning subscales.

Transgenders who were live with their parents had the better QoL in the role limitations due to mental health subscale, but transgenders who married after gender change and living with his/her spouse had highest QoL. Family support is one of the most important aspects affecting on transgender QoL and in line with the results of other studies [28, 36, 13].

Although this study had several results pertinent to the better understanding transgender QoL in Iranian transgenders, it has some limitations. The study is case-control and is limited in its ability to elucidate causal relationships. Our study was limited to transgenders who are referred to Isfahan and Shiraz counselling centers, thus, our data cannot be generalized to other transgender populations. The relatively small sample size in this study is a potential limitation. This study should therefore be repeated in larger samples. Lack of access to some transgenders after surgical intervention is another limitation of this study. The control group in this study was recruited from volunteers referred to counseling centers and may not be represent healthy individuals from the community. In particular, the control group was not matched for socio-demographic characteristics, although we matched for age and gender. However, this study offers new data from Iran, a developing country, which has been under-represented in past studies.

The findings suggest that service providers and policy makers urgently need to address the mental and physical health needs of transgenders, particularly younger ones.

## Conclusion

QoL of transgenders in subscales of physical functioning, social functioning, and role limitations due to physical function and vitality were lower than the general population. FTM transgender has better QoL than MTF. Employment, education, province of residence and economic status in addition to therapeutic intervention is associated with transgender’s QoL. The major finding of clinical importance is the poor QoL reported by transgenders compared to the control group, confirming the vulnerability of this population, and underlining the need for appropriate care and treatment.

## Abbreviations

ANOVA: Analysis of variance
FTM: Female to male
MTF: Male to female
QoL: Quality of life
SF-36: Short form 36
